# Digital Interventions for Older People Experiencing Homelessness: Systematic Scoping Review

**DOI:** 10.2196/63898

**Published:** 2025-02-21

**Authors:** Emily Adams, Eddie Donaghy, Caroline Sanders, Maria Klara Wolters, Lauren Ng, Christa St-Jean, Ryan Galan, Stewart William Mercer

**Affiliations:** 1 Advanced Care Research Centre Usher Institute University of Edinburgh Edinburgh United Kingdom; 2 Centre for Population Health Sciences Usher Institute University of Edinburgh Edinburgh United Kingdom; 3 Division of Population Health, Health Services Research, and Primary Care University of Manchester Manchester United Kingdom; 4 Informatics University of Edinburgh Edinburgh United Kingdom

**Keywords:** homeless, technology, digital exclusion, elderly, rough sleeping, digital intervention, older people, homelessness, systematic scoping review, aging, premature mortality, indicators, scoping review, databases, thematic analysis, telehealth, mhealth, ehealth

## Abstract

**Background:**

older people experiencing homelessness can have mental and physical indicators of aging several decades earlier than the general population and experience premature mortality due to age-related chronic conditions. Digital interventions could positively impact the health and well-being of homeless people. However, increased reliance on digital delivery may also perpetuate digital inequalities for socially excluded groups. The potential triple disadvantage of being older, homeless, and digitally excluded creates a uniquely problematic situation warranting further research. Few studies have synthesized available literature on digital interventions for older people experiencing homelessness.

**Objective:**

This scoping review examined the use, range, and nature of digital interventions available to older people experiencing homelessness and organizations supporting them.

**Methods:**

The scoping review followed Arksey and O’Malley’s proposed methodology, PRISMA-ScR (Preferred Reporting Items for Systematic Reviews and Meta-Analyses extension for Scoping Reviews) guidelines, and recent Joanna Briggs Institute guidelines. We searched 14 databases. Gray literature sources were searched to supplement the electronic database search. A narrative synthesis approach was conducted on the included articles, and common themes were identified inductively through thematic analysis.

**Results:**

A total of 19,915 records were identified through database and gray literature searching. We identified 10 articles reporting on digital interventions that had a clearly defined a participant age group of >50 years or a mean participant age of >50 years. A total of 9 of 10 studies were published in the United States. The study design included descriptive studies, uncontrolled pilot studies, and pilot randomized controlled trials. No studies aimed to deliver an intervention exclusively to older people experiencing homelessness or organizations that supported them. Four types of intervention were identified: telecare for people experiencing homelessness, distributing technology to enable digital inclusion, text message reminders, and interventions delivered digitally. Interventions delivered digitally included smoking cessation support, vocational training, physical activity promotion, and cognitive behavioral therapy. Overall, the included studies demonstrated evidence for the acceptability and feasibility of digital interventions for older people experiencing homelessness, and all 10 studies reported some improvements in digital inclusion or enhanced engagement among participants. However, several barriers to digital interventions were identified, particularly aspects related to digital inclusion, such as infrastructure, digital literacy, and age. Proposed facilitators for digital interventions included organizational and peer support.

**Conclusions:**

Our findings highlight a paucity of evaluated digital interventions targeted at older people experiencing homelessness. However, the included studies demonstrated evidence of the acceptability and feasibility of digital interventions for older people experiencing homelessness. Further research on digital interventions that provide services and support older people experiencing homelessness is required. Future interventions must address the barriers older people experiencing homelessness face when accessing digital technology with the input of those with lived experience of homelessness.

**Trial Registration:**

OSF Registries OSF.IO/7QGTY; https://doi.org/10.17605/OSF.IO/7QGTY

## Introduction

Homelessness is a complex phenomenon, with different conceptualizations making it challenging to establish its prevalence and study its phenomenology and effects. The European Observatory on Homelessness proposed the European Typology of Homelessness and Housing Exclusion (ETHOS) states homelessness can include rooflessness (without a shelter of any kind or sleeping rough), houselessness (with a place to sleep but temporarily in institutions or shelter), living in insecure housing (threatened with severe exclusion due to insecure tenancies, eviction, or domestic violence), and living in inadequate housing (in caravans on illegal campsites, in unfit housing, or in extreme overcrowding) [1]. People experiencing homelessness are thought to encounter “accelerated ageing” relative to the general population [2].

An interplay of health and social deprivation leads to people experiencing homelessness with disproportionately high rates of chronic illness [3] and premature age-adjusted mortality rates [4-6]. In this study, older people experiencing homelessness are defined as people older than 50 years who have experienced chronic/episodic homelessness. Chronic homelessness is associated with accelerated aging that predisposes younger people to geriatric health conditions normally associated with older than 75 years in the general population [7]. Older people experiencing homelessness are largely invisible in research, policy, and practice despite the rapidly increasing rates of this population [8]. In the United States, currently, half of single homeless adults are aged 50 and older, compared with 11% in 1990 [9,10]. Further, forecasts from US cohorts project significant growth in aged homelessness in age groups of 50 years and older and 65 years and older, revealing that much of the impact of the postwar baby boom on the aged homeless population is already well underway [11]. Similarly, in Scotland in 2022, 16% of new homeless applications were made by persons older than 50 years [12]. Consequently, the rapidly growing population of older people experiencing homelessness is of critical public health concern and warrants further research.

Homelessness is inextricably linked to social exclusion as individuals are often marginalized from participating in economic, political, social, and cultural life [13]. Concurrently, older people experiencing homelessness are particularly marginalized in the health care system. Older people experiencing homelessness face multiple barriers to timely and effective care for multiple long-term conditions [14,15]. The recent COVID-19 pandemic resulted in a shift from traditional face-to-face health care delivery toward an expansion in using digital technology for service provision [16,17]. In this study, we define digital interventions as interventions that incorporate using and accessing a digital device.

Digital interventions offer promising opportunities to explore new ways of intervention in harm reduction, well-being enhancement, and health treatment of older people experiencing homelessness [18]. This expansion of digital health care is positive for many; however, it has raised issues of digital inequalities for socially excluded groups, including physical barriers in a lack of access to equipment and educational barriers in not being able to use the technology [19]. This is of particular concern to older people experiencing homelessness as evidence has demonstrated that people older than 50 years experiencing homelessness have a lower prevalence of smartphone and internet access than adults aged older than 65 years in the general public or low-income adults [20]. Paradoxically, digital interventions hold new opportunities for inclusion for older people experiencing homelessness while presenting significant barriers due to unaddressed inhibited accessibility.

Over the past decade, more digital interventions have been used within homeless populations [19,21-23]. There is some existing evidence that older people experiencing homelessness meaningfully engage with technology [20,24]. However, no efforts have been made to synthesize this literature on digital interventions specifically for older people experiencing homelessness. Therefore, we conducted a scoping review to synthesize existing primary data from digital health interventions for older people experiencing homelessness. Our main research question was: what is the use, range, and nature of digital interventions available to older people experiencing homelessness and organizations that support people experiencing homelessness? To answer this research question, our scoping review aims to achieve the following objectives: (1) examine current digital interventions (delivery, implementation characteristics, and context) for people experiencing homelessness and the organizations that support them, (2) examine the use of included digital interventions by older people experiencing homelessness and organizations that support them, and (3) identify the facilitators and barriers for older people experiencing homelessness to inclusion in digital interventions.

The scoping review method was chosen because it provides a systematic, rigorous, and transparent approach to mapping a field of interest regarding existing research’s volume, nature, and characteristics [23]. Given that digital interventions available to older people experiencing homelessness are a rapidly developing area of research, a scoping review is an important first step in informing future research and practice [25].

## Methods

### Overview

This scoping review used the guidelines of Joanna Briggs Institute’s (JBI’s) Methodology for Scoping Reviews [26] and follows the methodological framework proposed by Arksey and O’Malley [27] which consists of the following stages: (1) identifying the research question; (2) identifying relevant studies; (3) selecting studies; (4) charting the data; and (5) collating, summarizing, and reporting the results. Our review also complies with the PRISMA-ScR (Preferred Reporting Items for Systematic Reviews and Meta-Analyses extension for Scoping Reviews) checklist (Multimedia Appendix 1) [25]. We first developed a scoping review protocol, including a rationale for conducting the review, the main objectives, search strategy, inclusion and exclusion criteria, and methods for screening and data extraction, that was then piloted and discussed by the research team before finalizing. The final protocol was registered retrospectively in Open Science Framework on May 15, 2023.

### Information Sources and Search Strategy

A systematic search strategy was developed in consultation with an expert librarian (RS). The search strategy also adhered to the Peer Review of Electronic Search Strategies (PRESS) guidelines [28]. We systematically searched 15 electronic databases from inception up to 28 July 2023: MEDLINE, Global Health, Cumulated Index to Nursing and Allied Health Literature (CINHAL), SCOPUS, APA PsycInfo, Embase, Academic Search Premier, International Bibliography of the Social Sciences (IBSS), Applied Social Sciences Index & Abstracts (ASSIA), Association for Computing Machinery Digital Library (ACMDL), Institute of Electrical and Electronics Engineers (IEEE), Web of Science, Educational Resources Information Centre (ERIC), and Cochrane library. Policy Commons was used to search for gray literature.

The systematic and comprehensive search strategy consisted of key search terms derived from existing search strings and bespoke for each electronic database. The search terms were as follows: homeless* OR temporary accommodation OR roofless OR unfit hous* OR inadequate hous* OR night shelter OR shelter* OR sofa surf* OR rough sleep* AND information communication technolog* OR cell phone* OR mobile app* OR mobile technolog* OR mobile health OR (m health OR e health OR mhealth or ehealth) OR online OR digital OR (telehealth OR tele health OR telemedicine OR tele medicine OR telecare OR tele care) OR social media OR internet OR (web based OR web-based) OR wearable* OR (Smartphone OR smart phone) OR Mobile phone OR Instant messag* OR (Email or electronic mail or e mail) OR (Smartwatch OR smart watch) OR (WhatsApp OR Instagram OR Facebook OR Telegram OR Signal OR Viber) (note: * indicates a wildcard). The results were combined using Boolean operations and adapted for each database (Multimedia Appendix 2). We also scanned references of the included articles for any relevant studies.

### Study Selection

After the publications were retrieved and duplicates removed using Endnote (Clarivate), search results were imported into the Covidence software management system (Veritas Health Innovation) for additional deduplication and screening by multiple reviewers.

As advised by JBI guidelines for conducting scoping reviews [26], the population, concept, and context framework was used to define eligibility. Textbox 1 shows the inclusion and exclusion criteria in line with the population, concept, and context framework and contains additional study elements relevant to the eligibility criteria. The ETHOS definition of homelessness framed the inclusion of participants experiencing homelessness [29]. Organizations supporting older people experiencing homelessness were considered to be any health or social care services or third-sector organizations providing a service to people experiencing homelessness. Due to accelerated aging, those who are 50 and experiencing homelessness are defined as “older” in contemporary research [2,30]. Therefore, only studies that included participants with a mean age of 50 years and older or studies with a clearly defined group of participants older than 50 years were included. The nature and causal pathways of homelessness vary globally [31]. To acknowledge that interventions for people experiencing homelessness will diverge due to social and cultural structures, health systems, and the provision of emergency accommodation [32], the scope of this review will be refined to solely high-income countries as defined by the Organization for Economic Co-operation and Development. This allowed for interventions to be more appropriately compared in the analysis [2]. So as not to exclude any innovative interventions, the definition of “digital intervention” was kept intentionally broad to include digital, web-based, or mobile interventions used by people experiencing homelessness to improve social, health, or prospective outcomes.

**Inclusion and exclusion criteria detailing the population, concept, and context framework for defining eligibility criteria for scoping reviews.**

**Inclusion criteria**

- Population: Participants currently absolutely homeless (living in shelters or on the streets) OR Participants currently in unstable housing situations (couch surfing, transiently housed) OR Organizations (health or social care services or third sector) that support the aforementioned participants AND Publications including people experiencing homelessness aged 50 years or older (mean participant age or clearly defined participant age group older than 50 years)
- Concept: Interventions are digital in delivery (web-based, mobile, applications, training, or social media)
- Context: Population in high-income nations or the Organization for Economic Co-operation and Development countries.
- Study type: Primary data presented

**Exclusion criteria**

- Population: Intervention not specific to homeless populations (eg, intervention for migrants or refugees)
- Concept: No digital intervention described OR Digital interventions solely designed for children or youth experiencing homelessness (younger than 18 years)
- Study type: No primary data presented

Further to the inclusion and exclusion criteria outlined in Textbox 1, throughout the review, publications were excluded for the following: (1) no full text available and (2) no English language version available.

Level 1 screening focused on inclusion criteria based on titles and abstracts, while level 2 screening involved reviewing full-text articles. Four reviewers (EA, LN, CS, and RG) independently screened all titles and abstracts. Reviewers met throughout the screening process to discuss queries and reduce uncertainties. Two reviewers (EA and LN) completed the full-text screening independently, with disagreements resolved by discussion with the reviewers and SWM.

### Charting Data and Reporting Results

The selected publications were read, annotated, and entered into a Microsoft Excel spreadsheet. EA and LN piloted the data extraction sheet with 2 of the included studies and then revised it in consultation with ED and SWM. We did not critically appraise the included studies, given that this is not typically an objective of a scoping review and the large research design heterogeneity of the studies reviewed [33].

To summarize the data, where applicable, we conducted a basic numerical analysis, for example, the proportion of participants older than 50 years in each study. Meta-analysis was not feasible due to the necessary inclusion of heterogeneous studies in answering the research question. We used a narrative synthesis approach to organize and present relevant findings. Qualitative data (eg, perceptions of digital interventions, barriers to digital interventions for older people experiencing homelessness, and facilitators to digital interventions for older people experiencing homelessness) were imported to NVivo 12 software (Lumivero) for analysis by EA. This approach is characterized by textual summaries and explanations of findings, which are first synthesized by thematic analysis to explore relationships among studies. Thematic analysis in this context involved iteratively identifying, classifying, and sorting the most important themes and concepts across studies [34]. The core research team (EA, LN, SWM, ED, MW, and CS) applied Braun and Clarke’s [35,36] reflexive thematic analysis approach, which involved familiarization with the data, generating initial codes, identifying and refining preliminary themes, reviewing themes collaboratively, and ultimately constructing and defining final themes. This iterative, reflexive process—guided by our own perspectives and the review’s research questions—enabled a deeper interpretation of the data, resulting in us developing the reported themes.

## Results

### Study Characteristics

**Figure 1:**
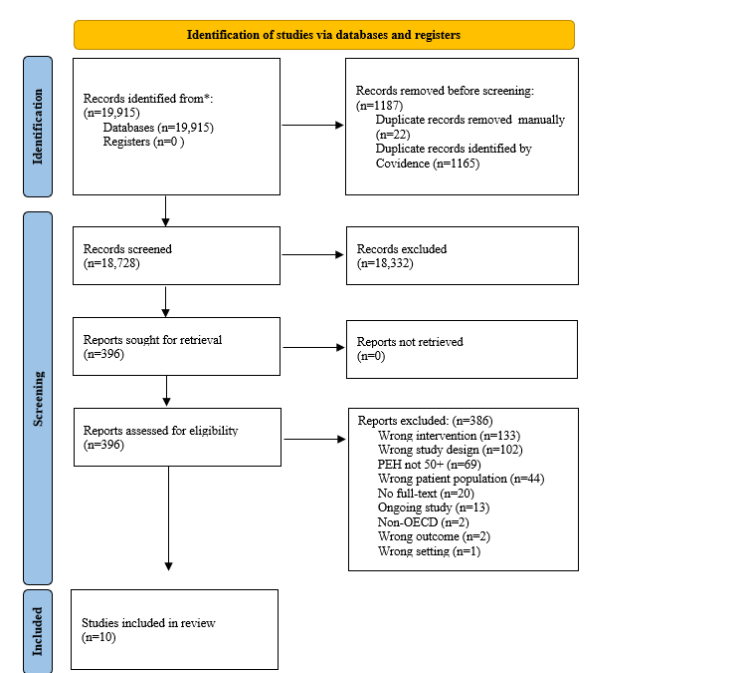
PRISMA-ScR (Preferred Reporting Items for Systematic Reviews and Meta-Analyses extension for Scoping Reviews) flow diagram for the identification of studies via databases [25]. OECD: Organization for Economic Co-operation and Development; PEH: people experiencing homelessness.

Our searches yielded 19,915 records. After removing duplicates 18,728 records were title and abstract screened (Multimedia Appendix 3). Ten articles met the inclusion criteria [37-46]. All records included in our review were peer-reviewed studies reporting on digital interventions for older people experiencing homelessness that had a clearly defined age group of participants older than 50 years or the mean participant age was older than 50 years (Figure 1 portrays the adapted PRISMA-ScR flow diagram). This screening criteria resulted in 90.5% (n=5902) of the total participants (N=6557) being older people experiencing homelessness (older than 50 years). In 6 of the studies, participants were veterans experiencing homelessness [38,39,41,42,45,46]. A total of 9 of the 10 studies were conducted in the United States [38-46], and the other was conducted in Hungary [37]. Studies were published between 2013 and 2023, with 4 published after the 2020 lockdowns of the COVID-19 pandemic [37,41,45,46]. The study design included descriptive studies [39,43], uncontrolled pilot studies [37,38,42,44,46], and pilot randomized controlled trials [40,41,45].

### Intervention Characteristics

**Figure 2:**
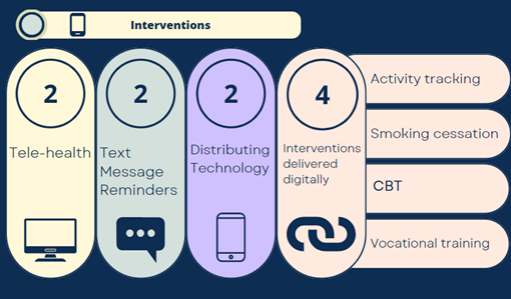
Summary of the 10 digital interventions included in this review. Interventions were categorized into implementing telecare for people experiencing homelessness, distributing technology to enable digital inclusion, text message reminder interventions, and 4 interventions delivered digitally. CBT: cognitive behavioral therapy.

The included studies were heterogeneous concerning intervention content, delivery, and reported outcomes. Tables 1 and 2 show the intervention delivery and implementation. Interventions could be categorized into implementing telecare for people experiencing homelessness [37,38], distributing technology to enable digital inclusion [39,46], text message reminder interventions [40,42], and 4 interventions delivered digitally [41,43-45] (summarized in Figure 2). Interventions delivered digitally ranged from smoking cessation support [43], vocational training [41], physical activity promotion [44], and cognitive behavioral therapy [45]. We used the broad definition of telecare used by Barlow et al [47], “the use of communications technology to provide health and social care directly to the user (‘patient’)” where interventions self-described as “telemedicine” [38] and “telehealth” and terms were used interchangeably.

**Table 1:** Summary of identified study and intervention characteristics.

Author, year	Sample size (% OPEH^a^, % Male)	Study design	Intervention	Intervention details
Békási et al, 2022 [37]	75 (100, 76)	Uncontrolled before and after (pre-post) pilot study	Telecare	Participants were invited to 6 web-based telecare visits biweekly with a focus on medical management of chronic conditions.
Gabrielian et al, 2013 [38]	14 (100, 71.4)	Uncontrolled mixed methods pilot study	Care Coordination Home Telehealth (CCHT)	CCHT used in-home messaging devices to provide health education and daily questions about clinical indicators from chronic illness care guidelines.
Garvin et al, 2021 [39]	1070 (97.1, 77.9)	Descriptive study	Tablet distribution	Veterans Association distributed tablets to access challenged veterans to be used for any clinical care that does not require physical contact (mental health therapy, medication management, primary care, palliative care, and rehabilitation care).
Kershaw et al, 2022 [40]	62 (100, 85)	Pilot randomized controlled trial (RCT)	Cell phone–based text messaging system	One-way message appointment reminders for upcoming appointments for a range of services and 2-way messages, which requested a text response, asked participants about their mood.
LePage et al, 2023 [41]	27 (81.5, 100)	Pilot developmental study and pilot RCT	Web-based vocational rehabilitation program	Manualized vocational program to aid individuals in identifying work skills, generating examples of those skills, and developing answers to typical questions one might encounter during the interview process.
McInnes et al, 2014 [42]	20 (80, 81)	Uncontrolled before and after pilot study	Text-messaging reminder intervention	Participants were sent 2 text message appointment reminders on a schedule of 5 days and 2 days before their appointment.
Reitzel et al, 2014 [43]	22 (100, 63.6)	Descriptive study	Smartphone-based Smoking cessation	The smartphone was programmed to collect latitude-longitude data via an internal GPS chip at the time the random assessment was prompted.
Rhoades et al, 2019 [44]	13 (100, 46.2)	Uncontrolled before and after pilot study	Cell phone-based physical activity intervention	Intervention to increase physical activity by encouraging walking via goal-setting and motivational text messaging, self-monitoring of walking behavior using pedometers, and providing ongoing feedback on walking performance.
Wilson et al, 2023 [45]	27 (100, 93)	RCT	Telephone-delivered cognitive behavioral therapy (CBT)	Telephone-delivered CBT, tobacco cessation pharmacotherapy, long-term incentives for abstinence-delivered counseling sessions, and optional prescribed tobacco cessation pharmacotherapy.
Wray et al, 2022 [46]	5127 (88, 87.8)	Uncontrolled before and after study	Distribution of video-enabled tablets and cell phones	Program that provided video-enabled tablets to any Veteran who was deemed to have necessary clinical services and a technological need.

^a^OPEH: older people experiencing homelessness.

**Table 2:** Summary of included digital intervention outcomes for older people experiencing homelessness.

Author, year	Duration (Months)	Follow-up	Outcomes measured	Main Findings
Békási et al, 2022 [37]	3	6 months post	Feasibility Patient experience Medical relevance	The study provided evidence of a feasible telecare setup in shelters for people experiencing homelessness. Client satisfaction was high; participants reported similarly high ratings for ease of use and comfort. Physicians reported the ability to assess the patient’s condition properly and make an adequate diagnosis.
Gabrielian et al, 2013 [38]	unclear	not stated	Program acceptability to staff and consumers Role of peers to support illness self-management	Participants were satisfied with CCHT^a^. Most did not require support from peers to engage in CCHT but valued peer social assistance.
Garvin et al, 2021 [39]	6	not stated	Tablet adoption and use	Tablet use was more common among veterans experiencing homelessness who were younger (AOR^b^=2.77; *P*<.001); middle-aged (AOR=2.28; *P*<.001); in rural. Use was less common among those who were Black (AOR=0.43; *P*<.001) and those with a substance use disorder (AOR=0.59; *P*<.001).
Kershaw et al, 2022 [40]	4	Immediately after (4 months)	Number of ED encounters Number of inpatient admissions Appointment keeping	No significant differences were found in ED admissions and inpatient or outpatient care between the intervention and control groups. Appointment no-show rates were 21.0% versus 30.6% (*P*=.08).
LePage et al, 2023 [41]	7 days	6 months post	The acceptability of the system The impact of the system	Veterans found the web-based program as acceptable as a hardcopy manual covering similar material. Participants randomized to the web-based system were more likely to obtain employment than people randomized to the hardcopy manual.
McInnes et al, 2014 [42]	2	Immediately after (2 months)	Feasibility, effectiveness, and acceptability of text message reminders for people experiencing homelessness	Participants were satisfied with the text-messaging intervention and had very few technical difficulties. Patient canceled visits, and no-shows trended downward.
Reitzel et al, 2014 [43]	1	6 months post	Associations between shelter proximity and real-time effects during a specific smoking quit attempt	Closer proximity to the shelter was associated with greater negative effects only during the post-quit attempt week (*P*=.008). All participants relapsed to smoking by 1-week post-quit attempt.
Rhoades et al, 2019 [44]	1.5	12 months post	Physical activity Acceptability Wellbeing	Changes to people’s physical activity levels were limited, but participants reported increased quality of life during the intervention period. Interviews revealed that the intervention was well-received and enjoyable for participants.
Wilson et al, 2023 [45]	1.5	3, 6, and 12 months post	The effectiveness of the intervention on biochemically verified prolonged smoking abstinence	At 6 months, participants in the mCM group were significantly more likely to meet the criteria for prolonged abstinence (AOR=3.1). Across time points, veterans in the mCM group had twice the odds of prolonged abstinence as those in the standard care group. However, by the 12-month follow-up, no statistically significant group difference in abstinence existed.
Wray et al, 2022 [46]	6	immediately (6 months post)	Characterize device recipients Assess in-person, telephone, and video-based engagement patterns across a variety of clinical settings	Compared with the 6 months prior to device receipt, in the 6 months following receipt, in-person and video engagement increased by an average of 1.4 visits (8%) and 3.4 visits (125%). Tablet users had a substantially more significant increase in video-based engagement (þ3.2 visits [þ110%] vs þ0.9 [þ64%]).

^a^CCHT: Care Coordination Home Telehealth.

^b^AOR: adjusted odds ratio.

No papers included aimed to develop or deliver interventions specifically for older people experiencing homelessness participants. Additionally, no interventions were found specifically for organizations supporting people experiencing homelessness. Thus, all included studies were developed to be used by individuals experiencing homelessness without specific considerations for older age.

### Outcomes From Digital Interventions

#### Overview

Thematic analysis of study outcomes identified 4 themes: improved digital inclusion, enhanced service engagement and care, no significant outcomes, and unintended consequences from digital interventions (summarized in Textbox 2). Individual intervention outcomes are summarized in Table 2. Barriers and facilitators for digital interventions were synthesized from the included interventions. Finally, the thematic analysis identified the overall feasibility of the included interventions.

**Outcomes from included digital interventions for older people experiencing homelessness and their impact on them.**

**Improved digital inclusion:**

- More equitable access to digital tools by improving the availability of resources [37-39,44,46]
- Improved perceptions of digital intervention [37,39]

**Enhancing service engagement and care:**

- Improvement in appointment attendance [40,42,46]
- Improvement in medication adherence [42]
- Improvement in perceived quality of care [37,38]

**Unintended outcomes:**

- Disengagement with in person services [38,46]

#### Improved Digital Inclusion

The outcomes of 5 included studies demonstrated more equitable access to digital tools for older people experiencing homelessness [37-39,44,46]. First, 2 studies evaluating device distribution programs concluded that tablet distribution offers a model for expanding access to health-related technology and telemedicine [39,46]. In the first study, Garvin et al [39] found that nearly half (45.9%) of veterans experiencing homelessness who received a tablet went on to use the device for video health consultation within 6 months of receipt. The most frequent tablet use was for tele-mental health support. In bivariate analyses, homeless tablet users were also less likely to have 3 or more chronic conditions (48.7% vs 56.7%; *P*=.006) or to have substance misuse disorder (47.6% vs 58.2%) [39]. The second study was a 6-month evaluation of people experiencing homelessness as digital device recipients. Wray et al [46] found engagement characteristics were similar between those who received a tablet or a cell phone, though fewer individuals with a cell phone had video encounters after receiving a device (45.3% vs 67.4%; *P*<.001), compared with those who received a tablet.

In addition to improving the availability of resources for digital inclusion, 2 studies addressed perceptions or acceptability of the intervention within the population, promoting digital inclusivity [37,39]. For example, during a 12-week telecare pilot for people experiencing homelessness in sheltered housing, Békási et al [37] demonstrated a significant difference in openness to telecare among people experiencing homelessness participants. They found that a group of previously digitally excluded homeless persons found the telecare visits helpful and valuable [37]. Similarly, Gabrielian et al [38] found support for telecare acceptability among homeless veterans with chronic conditions.

#### Enhancing Service Engagement and Care

Two studies used text message appointment reminders and showed improvement in appointment attendance and medication adherence for people experiencing homelessness [40,42]. In an 8-week pilot intervention period, McInnes et al [42] compared appointment attendance in pre and postintervention periods for a text messaging reminder intervention. They found that twice-weekly text message reminders led to a significant reduction of 30% in patient-cancelled appointments, and “no-shows” (missed appointments) were reduced by 19% [42]. Similarly, when they assessed the feasibility and effectiveness of text messaging to increase outpatient care engagement and medication adherence in people experiencing homelessness in Boston, Kershaw et al [40] recorded positive comments from participants overall. Qualitative findings from the follow-up interviews with intervention group participants showed that text messages functioned as social support. In addition, text messages complemented the participant’s lifestyle, and appointment reminders were helpful to ensure attendance [40].

The 2 telecare interventions demonstrated that digital delivery was acceptable to people experiencing homelessness and significantly improved the perceived quality of care [37,38]. Participants in the telecare pilot by Békási et al [37] were present at more than 90% of initially planned appointments, and almost 3-quarters of recruited clients completed the whole course of 6 web-based visits. In postintervention qualitative interviews for a Care Coordination Home Telehealth intervention, participants described telecare as user-friendly and promoted illness self-management [11]. Similarly, Wray et al [46] found that the distribution of “tablets” to veterans improved participants’ access to clinical services as it facilitated telecare uptake.

Three digital interventions improved the health and well-being outcomes of people experiencing homelessness [40,44,45]. In a comparative effectiveness trial of digital smoking cessation, Wilson et al [45] reported that veterans in the digitally delivered cognitive behavioral therapy group had twice the odds of prolonged tobacco abstinence compared with the control. Similarly, Rhoades et al [44] identified text messaging and the use of pedometers as a feasible and promising option for improving health and well-being among people experiencing homelessness, as slightly more than half (54%) of participants increased their weekly steps from the beginning to the end of the intervention.

#### Digital Interventions Demonstrated no Significant Difference

Conversely, 2 studies could not demonstrate any significant difference between control and intervention groups in prestated outcomes for some aspects of digital interventions [40,41]. Kershaw et al [40] aimed to demonstrate outcomes in the text messaging reminders that impacted inpatient care for veterans experiencing homelessness; however, no significant differences were found in an inpatient or outpatient care engagement between the intervention and control groups. Additionally, when stratified by appointment type, there were no significant differences in appointment keeping between intervention and control groups, and estimated effect sizes were small for both appointment types. However, effect sizes for completed appointments and no-shows were slightly larger for physical health appointments than for behavioral health appointments [40]. In a randomized controlled pilot test of employment outcomes, Lepage et al [41] found that the web-based vocational rehabilitation program’s control and intervention groups did not differ significantly in the number of modules completed.

#### Unintended Outcomes of Digital Interventions

Two studies reported unintended consequences of implementing digital interventions [38,46]. Wray et al [46] observed a “substitutive effect”—where telephone-based engagement decreased while in-person and video-based engagement increased at a commensurate rate. Further, compared with those who received a cell phone, those who received a tablet had a smaller increase in in-person (1.3 visits, 8% vs 2.1 visits, 13%) visits and a greater decrease (4.6 visits, 23% vs 1.8 visits, 12%) in telephone visits. They suggest this could negatively impact patients’ willingness to engage with in-person care options [46]. This aligns with the findings of Gabrielian et al [38], which reported the unintended negative consequence of participants feeling detached from the technology by the digital delivery of telehealth. In particular, participants felt digital delivery was impersonal.

### Barriers and Facilitators to Digital Interventions for Older People Experiencing Homelessness

#### Overview

Five studies reported age and pre-existing digital exclusion as barriers to people experiencing homelessness in the studied digital interventions [37,39,40,42,46]. Table 3 summarizes the barriers and facilitators identified in the review. Three studies found that the predominant facilitators of digital interventions for people experiencing homelessness were organizational support and peer support [37,39,44].

**Table 3:** Summary of the reported barriers and facilitators of digital interventions of older people experiencing homelessness.

Barriers	Facilitators
Digital literacy [40,41]	Organization support/technical assistance [37,40,44,46]
Device loss and theft [40]	Charging spots [40]
Internet connection [46]	Peer support [38]
Prohibitive cost (data and minutes) [37,42]	—^a^
Age [39]	—

^a^Not applicable.

#### Barrier: Digital Exclusionary Factors for People Experiencing Homelessness

Three studies noted participants’ difficulties in operating devices and a need for supporting digital literacy. Kershaw et al [40] highlighted digital literacy problems, with 31% (n=19) of participants reporting some technical difficulty, confusion, and usability problems when operating flip phones. Furthermore, Kershaw et al [40] reported loss and theft of mobile devices frequently during the study, requiring numerous replacements given the population is at elevated risk of experiencing theft and limited ability to store devices securely. In distributing tablets to people experiencing homelessness, Wray et al [46] highlighted challenges in maintaining connectivity to the internet and digital literacy problems as factors negatively impacting their experience of such tools. Similarly, McInnes et al [42] noted financial barriers to mobile phone use (eg, running out of minutes).

#### Barrier: Age

One study reported age as a barrier to the tablet adoption intervention. Following distribution, tablet use was more common among veterans experiencing homelessness who were younger (adjusted odds ratio 2.77; *P*<.001). Garvin et al [39] suggest that older veterans would benefit from simplified user interface designs and digital literacy training to increase comfort, confidence, and willingness to use.

#### Facilitator: Organizational Support Required

No studies identified in the review focused their intervention on organizations supporting people experiencing homelessness. However, 4 studies note that assistance from support staff or research teams was required in the set-up or duration of the intervention [37,40,44,46]. In the telecare intervention of Békási et al [37], the presence of on-site assistants served as technical support and prevented any misunderstandings regarding medication or referral issues. Rhoades et al [44] gave participants one-on-one assistance with using text messaging as needed. Wray et al [46] preconfigured devices and loaded them with videoconferencing software and mobile apps for participant ease. Kershaw et al [40] conclude that making charging more readily available where homeless persons spend their time could also help reduce theft, such as near bedsides in shelters (inside lockers) and more widely available in libraries, health clinics, food banks, and soup kitchens.

#### Facilitator: Peer Support

Two studies suggested implementing peer support to enhance adherence and troubleshoot utility issues [38,45]. Gabrielian et al [38] employed peer mentors to conduct visits with veterans to assess relevant psychosocial circumstances and report back to researchers on any equipment/medical concerns. They suggested that peers could significantly break down patient-level barriers to participation in telecare management. Nevertheless, institutional obstacles prevented peer contact with veterans- with 10 of 14 participants opting for adjunctive peer support [38]. Similarly, Wilson et al [45] suggest approaches that integrate peer support into smoking cessation intervention sessions/groups might be beneficial, given previous research indicating that knowing 5 quitters was associated with greater odds of achieving smoking abstinence among homeless smokers.

### Potential Viability of Digital Interventions for Older People Experiencing Homelessness

Three included studies concluded that the intervention demonstrated the viability of digital delivery for older people experiencing homelessness [37,39,42]. There was evidence of a feasible telecare setup in shelters offering accommodation to people experiencing homelessness that might support the planning of future telecare services for vulnerable populations [37]. McInnes et al [42] proposed that text messages are a feasible digital intervention as they are an unobtrusive connection to patients, and mobile phones are one of the few communication tools that people experiencing homelessness can attain. Further, they concluded that appointment reminders are greatly needed for this population because they frequently lack the tools that nonhomeless take for granted: reliable mailing addresses, landline phones, wall or computerized calendars, and social supports to remind them of appointments [42].

## Discussion

### Principal Findings

The scoping review examined the range, nature, and use of digital interventions available to older people experiencing homelessness and organizations that support older people experiencing homelessness. We identified only concerned studies within an Organization for Economic Co-operation and Development context detailing digital interventions with participants who were older and experiencing homelessness. The nature of interventions included digitally delivered primary health care (telecare) [37,39], appointment reminders [40,42], technology distribution [39,46], and well-being interventions delivered in a digital format [41,43-45]. Included interventions found that common barriers were existing digital exclusion factors for older people experiencing homelessness, such as digital literacy, absence of safe storage for technology, and inconsistent internet connectivity. Suggested facilitators for older people experiencing homelessness in digital interventions were organizational and peer support. The searches found no interventions designed for adoption by support services (as opposed to the older people experiencing homelessness user). Further, it should be noted there was a lack of studies reporting on other dimensions of exclusion (ethnicity, gender, etc) for older people experiencing homelessness. Finally, no included interventions were intended for sole use by a cohort of older homeless adults; subsequently, there were no specific considerations for older people in the intervention design. Therefore, this review highlights the paucity of digital interventions designed for and delivered to older people experiencing homelessness.

### Older People Experiencing Homelessness and Digital Engagement

This review demonstrates evidence that digital interventions could benefit older people experiencing homelessness. Consequently, it is crucial to understand the prevalence and use of technology among older people experiencing homelessness to implement digital interventions effectively. People experiencing homelessness access to mobile devices varies greatly. One study showed that as many as 95% had a mobile phone, and 77% reported having a smartphone [48]. However, participants from an ongoing clinical trial at a homeless shelter in Texas reported lower (28.4%) access to an active cell phone. However, 88.6% of participants reported at least weekly internet use, and 77.2% used email [49]. It is well established that older adults in the general population use technological solutions at lower rates than younger adults [50]. In addition, little is known about access to and use of the internet and mobile phones by older homeless adults. Raven et al [20] make one of the only attempts to describe the access to and use of mobile phones, computers, and the internet among 350 homeless adults older than 50 years. They found that most (72.3%) participants owned or had mobile phone access. Participants used phones and the internet to communicate with medical personnel (64.6%), search for housing and employment (30.7%), and to contact their families (82.3%). They concluded that participants had a lower prevalence of smartphone and internet access than adults aged older than 65 years in the general public or low-income adults. Participants faced barriers to mobile phone and internet use, including financial barriers and functional and cognitive impairments [20].

Only one included intervention analyzed age as a variable for use, where tablet use was less likely for older participants [39]. Similarly, in a sample of homeless-experienced adults aged 50 years and older, Raven et al [20] found that almost 3-quarters of participants had current access to a mobile phone. However, participants had a lower prevalence of smartphone and internet access than adults older than 65 years in the general public or low-income adults [20]. This demonstrates that premature aging and complex social challenges that are attributed to homelessness are significant factors in digital exclusion. Concurrently, in the literature, there are significantly more evaluated digital interventions for youth experiencing homelessness (YEH) than those retrieved for this review [49,51-61]. The disparity in tailored interventions for YEH and older people experiencing homelessness further illustrates the widening digital divide for older people experiencing homelessness.

### Digital Exclusion as a Barrier

Three included studies highlighted exclusionary factors associated with homelessness, causing barriers for digital interventions [40,42,46]. Technological competency, limited safe storage, and lack of internet connectivity were all referenced in this review as barriers to digital interventions for individuals experiencing homelessness [40,46]. Sieck et al [62] state that digital literacies and internet connectivity have been called the “super social determinants of health” because they address all other social determinants of health. For example, applications for employment, housing, and other assistance programs, are increasingly, and sometimes exclusively, accessible via the web [62]. In their systematic review of technology for people experiencing homelessness, Heaslip et al [19] found that older people experiencing homelessness felt further marginalized by the modern benefits system that “assumes” digital competence and confidence. As participation in most included studies was voluntary, older people experiencing homelessness with a more positive attitude and openness toward telecare might have been overrepresented in the sample, skewing the sample to those more digitally literate [37]. “Access instability” is a term used by Galperin et al [63] to describe their findings that reliable access to electrical power represents a fundamental yet understudied barrier to mobile use. Lacking a safe and reliable place to charge devices, the unstably housed must activate coping strategies that limit digital engagement and constrain use [63]. Overall, this suggests that while digital interventions have the potential to expand inclusion, existing literacy and connectivity barriers must be addressed in tandem with implementation.

### Facilitators of Digital Interventions

Despite those barriers, digital interventions can also facilitate access to care. In this review, Garvin et al [39] suggest older veterans would benefit from simplified computer app designs and digital literacy training to increase comfort, confidence, and willingness to engage in their tablet adoption intervention. Concurrently, Sieck et al [62] posit that improving digital literacy skills to access valuable digital tools is key to reducing disparities. In a digital access survey for people experiencing homelessness, Sturman et al [64] found that digital literacy was positively associated with uptake in digital health interventions. Relatedly, Van den Berk-Clark and McGuire [65] argue that the issue of trust between people experiencing homelessness and support services is multifaceted and is influenced by technical competence and the degree to which the individual is made to feel welcome. A study of video consultation suggested that clinicians interacting with homeless-experienced older adults should prioritize addressing the potential skepticism of video calls. Further, it is proposed that clinicians should assess their access to and knowledge of video conferencing technology [66].

Similarly, 4 studies found that organizational or research team support facilitated the engagement of people experiencing homelessness with digital interventions. Furthermore, the authors identified peer support as a key contributor to participants’ comfort with digital interventions [37,40,44,46]. However, Gabrielian et al [38] stated that most participants did not use peer support and highlighted that fostering trust with those providing technology assistance was of primary importance. Similarly, Glover et al [54] found that YEH preferred automated mobile phone functions that avoided interaction with professionals and peers.

This review included 6 studies with interventions conducted with veterans in the United States [38,39,41,42,45,46]. The interventions benefited from the support and infrastructure of the US Department of Veterans Affairs (VA) [67]. It can be assumed that the integration of VA into the health system facilitated the implementation of the interventions. However, additional evaluative research on the context of the implementation is required to determine if internal process barriers nullify any potential facilitatory effects [68].

### Implications for Policy and Practice and Future Research

This scoping review highlights several research gaps, upon which we base the following recommendations:

1. The review demonstrated a largely positive view of older people experiencing homelessness’s digital interventions however, this needs to be supported by additional empirical evidence of the health and well-being benefits.
2. Additional research is needed to examine older people experiencing homelessness’s access to and use of digital tools and interventions.
3. More research is needed on the digital health literacy skills of older people experiencing homelessness and their experiences of using technology to search for and access information and services.
4. Additional evaluation of the implementation infrastructure, for example, the health system deploying a telecare initiative, on the efficacy of an intervention for older people experiencing homelessness
5. Finally, future research should also focus on developing and evaluating digital interventions for older people experiencing homelessness.

The use of digital interventions is a rapidly developing area of practice for professionals with several elements to consider, including:

1. increasing access to technology
2. optimizing technology-based infrastructure,
3. providing training for community outreach and practitioners,
4. engaging service users in the co-design of diverse and contextually sensitive interventions

Due to longstanding digital barriers, implementing digital interventions without addressing older people experiencing homelessness’s digital exclusion will likely further damage trust and perpetuate existing poor support service access. As such, services should:

5.     systematically assess individual patients’ digital literacies,

6.     learn about their internet access, and

7.     work to address their needs.

8.     Partner with community-based organizations with expertise in digital literacy training to address comfortability.

Policy for digital inclusion of older people experiencing homelessness should prioritize free and accessible technology in public settings (eg, shelters, community centers, libraries, and harm reduction centers) and free access to mobile devices. Action is needed across government, public, private, and third-sector organizations to ensure capitalization on the potential for digital interventions to address health and well-being while minimizing the risk of exacerbating existing health inequalities.

### Strengths and Limitations

This scoping review has several strengths. To the best of our knowledge, it is the first scoping review to describe the digital interventions for older people experiencing homelessness. We followed the JBI guidance for scoping reviews [33] and our database searching, handling of data, and reporting adhered to published guidelines for undertaking a robust standard scoping review [25,27].

Several limitations should be highlighted. Given the heterogeneity of study methods, we did not systematically assess the quality of studies. Similarly, only English language publications were included due to time and human resources. As there was inconsistency in how papers reported participant age, either a subgroup of participants was clearly defined as older than 50 years or the entire participant group’s mean age was older than 50 years. We acknowledge that this inclusion method may generate an incomplete picture of available interventions for older people experiencing homelessness. Most participants in the included studies are veterans based in the United States; therefore, any attempt to generalize these results should be undertaken with caution.

Finally, as this study is not a formal meta-analysis, we did not use more complex statistical pooling methods or analyze the heterogeneity in outcomes reported; as such, our results should be interpreted with these considerations in mind.

### Conclusion

Our findings demonstrate the paucity of bespoke digital interventions for older people experiencing homelessness. However, the included studies demonstrate some evidence for the acceptability and feasibility of digital interventions for older people experiencing homelessness. To leverage the potential benefits of digital interventions for older people experiencing homelessness, implementing such interventions will require additional consideration of the multiple exclusionary factors experienced by older people experiencing homelessness. The anticipated increase in the number of older people experiencing homelessness warrants further research on the impact of digital interventions for this vulnerable population.

## Appendix

Multimedia Appendix 1 PRISMA-ScR (Preferred Reporting Items for Systematic Reviews and Meta-Analyses extension for Scoping Reviews) checklist.

Multimedia Appendix 2 Search Strings.

Multimedia Appendix 3 Database returned results.

## Abbreviations

ACMDL: Association for Computing Machinery Digital Library
ASSIA: Applied Social Sciences Index & Abstracts
CINHAL: Cumulated Index to Nursing and Allied Health Literature
ERIC: Educational Resources Information Centre
ETHOS: European Typology of Homelessness and Housing Exclusion
IBSS: International Bibliography of the Social Sciences
IEEE: Institute of Electrical and Electronics Engineers
JBI: Joanna Briggs Institute
PRESS: Peer Review of Electronic Search Strategies
PRISMA-ScR: Preferred Reporting Items for Systematic Reviews and Meta-Analyses extension for Scoping Reviews
VA: Veterans Affairs
YEH: youth experiencing homelessness
