# Supplementary material for: Digital Interventions for Older People Experiencing Homelessness: Systematic Scoping Review
Source: J Med Internet Res. 2025 Feb 21;27:e63898. doi: 10.2196/63898 (PMC11890140; doi:10.2196/63898)
Supplement: Multimedia Appendix 2 [file jmir_v27i1e63898_app2.docx]

**PCC framework**

| **Participants** | Older People Experiencing Homelessness (OPEH) |
| --- | --- |
| **Concept** | Digital interventions |
| **Context** | High income countries |

**PCC definitions**

| **PCC** | **This review** | **Definition sources** | **My definition** |
| --- | --- | --- | --- |
| **Participants** | Older People Experiencing Homelessness (OPEH) | 1. *Homelessness*    European Typology of Homelessness and housing exclusion (ETHOS) (Edgar, 2012)  2. *OPEH*  OPEH include people aged 50+ who have experienced chronic/episodic homelessness or are experiencing homelessness for the first time in later life, both of which are associated with accelerated ageing that predisposes younger-aged people to geriatric health conditions normally associated with old age (Brown et al., 2013) | **OPEH-** People with multiple complex needs OR over 50 years AND experiencing homelessness according to ETHOS definition (Edgar, 2012)  OR  **Organisations that support OPEH**- any health or social care services or third sector organisations providing support or signposting to PEH. |
| **Concept** | Digital interventions | A Digital Public Health Intervention addresses at least one essential Public Health function through digital means. Applying a framework for functional classification and stratification categorizes its interaction level with the user. The developmental process of a digital public health intervention includes the user perspective by applying participatory methods to support its effectiveness and implementation with the goal to achieve a population health impact. (Wienert et al., 2022) | **Digital interventions-** Any form of digital, online or mobile intervention used by PEH to improve social, health or prospective outcomes |
| **Context** | High-income countries | OECD | Filtered in screening |

**Databases**

| **Elements for search saturations** | **Database** | **Interface** |
| --- | --- | --- |
| **PCC** | | |
| **PEH** | **Cochrane library** (Cochrane systematic reviews) | Cochrane Library  https://www-cochranelibrary-com.ezproxy.is.ed.ac.uk/ |
|  | **Medline***Insert specific selection/Date/etc* | [Ovid](https://ovidsp-dc1-ovid-com.ezproxy.is.ed.ac.uk/ovid-b/ovidweb.cgi?QS2=434f4e1a73d37e8c795e9e0ca985fabc8391dd377045fe09eadff12f414b5332f332e07532f9a74c6d1aafa32146d56f378d4d2a5ef91609aa8268f4577d7e4509dac5db464c2fe4566db15816073ef460a05dd0b3d2570395f2fde0b0253e3bd86d41e6a3ffdb2ef556ce56ebc1b9cabe8ccd3106a4b5ad160493a9752895bd0f51613a65715ba8b746d307fc6e7522e8369ffb3f911610b7fae6ce2663a219e3a62fb6cc61ea0770bf08b511b318132f4fff1e387d3663337b2101f8a994d51acef20e61cda865) |
|  | **Global Health** *not achieve * | [Ovid](https://ovidsp-dc1-ovid-com.ezproxy.is.ed.ac.uk/ovid-b/ovidweb.cgi?QS2=434f4e1a73d37e8c795e9e0ca985fabc8391dd377045fe09eadff12f414b5332f332e07532f9a74c6d1aafa32146d56f378d4d2a5ef91609aa8268f4577d7e4509dac5db464c2fe4566db15816073ef460a05dd0b3d2570395f2fde0b0253e3bd86d41e6a3ffdb2ef556ce56ebc1b9cabe8ccd3106a4b5ad160493a9752895bd0f51613a65715ba8b746d307fc6e7522e8369ffb3f911610b7fae6ce2663a219e3a62fb6cc61ea0770bf08b511b318132f4fff1e387d3663337b2101f8a994d51acef20e61cda865) |
|  | **CINHAL** | [**EBSCOhost**](https://web-p-ebscohost-com.ezproxy.is.ed.ac.uk/ehost/search/advanced?vid=0&sid=a480910c-d727-421d-a17d-df467af85359%40redis) |
|  | **SCOPUS *abstract and key word*** | [**Scopus**](https://www-scopus-com.ezproxy.is.ed.ac.uk/search/form.uri?display=advanced) |
|  | **APA PsychInfo** | [Ovid](https://ovidsp-dc1-ovid-com.ezproxy.is.ed.ac.uk/ovid-b/ovidweb.cgi?QS2=434f4e1a73d37e8c795e9e0ca985fabc8391dd377045fe09eadff12f414b5332f332e07532f9a74c6d1aafa32146d56f378d4d2a5ef91609aa8268f4577d7e4509dac5db464c2fe4566db15816073ef460a05dd0b3d2570395f2fde0b0253e3bd86d41e6a3ffdb2ef556ce56ebc1b9cabe8ccd3106a4b5ad160493a9752895bd0f51613a65715ba8b746d307fc6e7522e8369ffb3f911610b7fae6ce2663a219e3a62fb6cc61ea0770bf08b511b318132f4fff1e387d3663337b2101f8a994d51acef20e61cda865) |
|  | **Embase** | [Ovid](https://ovidsp-dc1-ovid-com.ezproxy.is.ed.ac.uk/ovid-b/ovidweb.cgi?QS2=434f4e1a73d37e8c795e9e0ca985fabc8391dd377045fe09eadff12f414b5332f332e07532f9a74c6d1aafa32146d56f378d4d2a5ef91609aa8268f4577d7e4509dac5db464c2fe4566db15816073ef460a05dd0b3d2570395f2fde0b0253e3bd86d41e6a3ffdb2ef556ce56ebc1b9cabe8ccd3106a4b5ad160493a9752895bd0f51613a65715ba8b746d307fc6e7522e8369ffb3f911610b7fae6ce2663a219e3a62fb6cc61ea0770bf08b511b318132f4fff1e387d3663337b2101f8a994d51acef20e61cda865) |
|  | **Academic Search Premier** | [**EBSCOhost**](https://web-p-ebscohost-com.ezproxy.is.ed.ac.uk/ehost/search/advanced?vid=0&sid=a480910c-d727-421d-a17d-df467af85359%40redis) |
|  | **International Bibliography of the Social Sciences (IBSS)‎** | [**ProQuest**](https://www.proquest.com/ibss/databases/index?accountid=10673) |
|  | [**Applied Social Sciences Index & Abstracts (ASSIA)‎**](https://www.proquest.com/assia/socialsciences/fromDatabasesLayer?accountid=10673) | [**ProQuest**](https://www.proquest.com/ibss/databases/index?accountid=10673) |
| **Digital Interventions** | **ACMDL Association for Computing Machinery Digital Library (ACMDL)- *abstracts*** | **https://dl-acm-org.ezproxy.is.ed.ac.uk/** |
|  | **Institute of Electrical and Electronics Engineers (IEEE)** | **https://ieeexplore-ieee-org.ezproxy.is.ed.ac.uk/Xplore/home.jsp** |
|  | **Web of Science** | [Document search - Web of Science Core Collection (ed.ac.uk)](https://www-webofscience-com.ezproxy.is.ed.ac.uk/wos/woscc/basic-search) |
|  | **Educational Resources Information Centre (ERIC)** | [**EBSCOhost**](https://web-p-ebscohost-com.ezproxy.is.ed.ac.uk/ehost/search/advanced?vid=0&sid=a480910c-d727-421d-a17d-df467af85359%40redis) |
| **Other sources** | | |
| **Grey literature** | **Policy Commons** | [**https://policycommons-net.ezproxy.is.ed.ac.uk/**](https://policycommons-net.ezproxy.is.ed.ac.uk/) |

Search Strings

*Supplementary excel document with search results)

| **Interface** | **Databases** | **Search string** |
| --- | --- | --- |
| Ovid | APA PsychInfo | homeless* OR temporary accommodation OR roofless OR unfit hous* OR inadequate hous* OR night shelter OR shelter* OR sofa surf* OR rough sleep*  AND   information communication technolog* OR  cell phone* OR mobile app* OR mobile technolog* OR mobile healt OR  (m health OR e health OR mhealth or ehealth) OR online OR digital OR (telehealth OR tele health OR telemedicine OR tele medicine OR telecare OR tele care) OR social media OR internet OR (web based OR web-based) OR wearable* OR (Smartphone OR smart phone) OR Mobile phone OR Instant messag* OR (Email or electronic mail or e mail) OR (Smartwatch OR smart watch) OR (WhatsApp OR Instagram OR Facebook OR Telegram OR Signal OR Viber) |
|  | Embase |  |
|  | Medline |  |
|  | Global Health |  |

| **Interface** | **Databases** | **Search string** |
| --- | --- | --- |
| EBSCOhost | CINHAL | AB homeless* OR temporary accommodation OR roofless OR unfit hous* OR inadequate hous* OR night shelter OR shelter* OR sofa surf* OR rough sleep*  AND  AB information communication technolog* OR cell phone* OR mobile app* OR mobile technolog* OR mobile health OR (m health OR e health OR mhealth or ehealth) OR online OR digital OR (telehealth OR tele health OR telemedicine OR tele medicine OR telecare OR tele care) OR social media OR internet OR (web based OR web-based) OR wearable* OR (Smartphone OR smart phone) OR Mobile phone OR Instant messag* OR (Email or electronic mail or e mail) OR (Smartwatch OR smart watch) OR (WhatsApp OR Instagram OR Facebook OR Telegram OR Signal OR Viber) |
|  | Academic Search Premier |  |
|  | Educational Resources Information Centre (ERIC) |  |

| **Interface** | **Databases** | **Search string** |
| --- | --- | --- |
| ProQuest | International Bibliography of the Social Sciences (IBSS)‎ | noft(homeless* OR temporary accommodation OR roofless OR unfit hous* OR inadequate hous* OR night shelter OR shelter* OR sofa surf* OR rough sleep*) AND noft(information communication technolog* OR cell phone* OR mobile app* OR mobile technolog* OR mobile health OR (m health OR e health OR mhealth OR ehealth) OR online OR digital OR (telehealth OR tele health OR telemedicine OR tele medicine OR telecare OR tele care) OR social media OR internet OR (web based OR web-based) OR wearable* OR (Smartphone OR smart phone) OR Mobile phone OR Instant messag* OR (Email OR electronic mail OR e mail) OR (Smartwatch OR smart watch) OR (WhatsApp OR Instagram OR Facebook OR Telegram OR Signal OR Viber)) |
|  | Applied Social Sciences Index & Abstracts (ASSIA)‎ |  |

| **Databases** | **Search string** |
| --- | --- |
| ACM | Abstract: homeless*] OR [Abstract: shelter*] |

| **Databases** | **Search string** |
| --- | --- |
| IEEE | ("All Metadata":homeless* OR "All Metadata":temporary accommodation OR "All Metadata":roofless OR "All Metadata":unfit hous* OR "All Metadata":inadequate hous* OR "All Metadata":night shelter OR "All Metadata":shelter* OR "All Metadata":sofa surf* OR "All Metadata":rough sleep*) |

| **Databases** | **Search string** |
| --- | --- |
| Web of Science | [Homeless* OR temporary accommodation OR roofless OR unfit hous* OR inadequate hous* OR night shelter OR shelter* OR sofa surf* OR rough sleep*] AND [information communication technolog* OR cell phone* OR mobile app* OR mobile technolog* OR mobile health OR (m health OR e health OR mhealth or ehealth) OR online OR digital OR (telehealth OR tele health OR telemedicine OR tele medicine OR telecare OR tele care) OR social media OR internet OR (web based OR web-based) OR wearable* OR (Smartphone OR smart phone) OR Mobile phone] |

| **Databases** | **Search string** |
| --- | --- |
| Scopus | homeless* AND information AND communication AND technolog* OR cell AND phone* OR mobile AND app* OR mobile AND technolog* OR mobile AND health OR m AND health OR e AND health OR mhealth OR ehealth OR online OR digital OR telehealth OR tele AND health OR telemedicine OR tele AND medicine OR telecare OR tele AND care OR social AND media OR internet OR web AND based OR web-based OR wearable* OR smartphone OR smart AND phone OR mobile AND phone |

| **Databases** | **Search string** |
| --- | --- |
| Policy Commons | summary:homeless* AND summary:digital |

| **Databases** | **Search string** |
| --- | --- |
| Cochrane Library | (homeless*):ti,ab,kw AND (nformation communication technolog* OR cell phone* OR mobile app* OR mobile technolog* OR mobile health OR (m health OR e health OR mhealth or ehealth) OR online OR digital OR (telehealth OR tele health OR telemedicine OR tele medicine OR telecare OR tele care) OR social media OR internet OR (web based OR web-based) OR wearable* OR (Smartphone OR smart phone) OR Mobile phone OR Instant messag* OR (Email or electronic mail or e mail) OR (Smartwatch OR smart watch) OR (WhatsApp OR Instagram OR Facebook OR Telegram OR Signal OR Viber)):ti,ab,kw (Word variations have been searched) |
